# Association of Biologic/Targeted-Synthetic DMARDs with a Lower Prevalence of Hand Joint Deformity in Rheumatoid Arthritis: A Cross-Sectional Real-World Study

**DOI:** 10.3390/medicina62020241

**Published:** 2026-01-23

**Authors:** Ying Yang, Jian-Zi Lin, Yao-Wei Zou, Ya-Nan Cao, Tao Wu, Pei-Yu Lin, Ran Shi, Zhi-Ming Ouyang, Kui-Min Yang, Ze-Hong Yang, Jian-Da Ma, Lie Dai

**Affiliations:** 1 Department of Rheumatology and Immunology, Sun Yat-Sen Memorial Hospital, Sun Yat-Sen University, 107 Yan Jiang West Road, Guangzhou 510120, China; yangy935@mail2.sysu.edu.cn (Y.Y.); linjz5@mail.sysu.edu.cn (J.-Z.L.); zouyw8@mail.sysu.edu.cn (Y.-W.Z.); caoyn6@mail2.sysu.edu.cn (Y.-N.C.); wutao68@mail.sysu.edu.cn (T.W.); lampu@mail2.sysu.edu.cn (P.-Y.L.); shir29@mail2.sysu.edu.cn (R.S.); ouyzhm3@mail.sysu.edu.cn (Z.-M.O.); yangkm5@mail.sysu.edu.cn (K.-M.Y.); 2 Department of Radiology, Sun Yat-Sen Memorial Hospital, Sun Yat-Sen University, 107 Yan Jiang West Road, Guangzhou 510120, China; yangzeh2@mail.sysu.edu.cn

**Keywords:** rheumatoid arthritis, hand joint deformity, biologic/targeted synthetic DMARDs

## Abstract

*Background and Objectives*: Hand joint deformity remains a main cause impairing quality of life in rheumatoid arthritis (RA). This study aimed to investigate the association between biologic and targeted-synthetic disease-modifying antirheumatic drugs (b/tsDMARDs) treatment and the prevalence of hand joint deformity in RA patients. *Materials and Methods*: This cross-sectional analysis included RA patients recruited between 2019 and 2024. Hand joint deformity was defined as the presence of specific deformity in any of 28 hand joints, including the metacarpophalangeal (MCP) joints I-V, proximal interphalangeal (PIP) joints I-V, and distal interphalangeal (DIP) joints II-V. The key exposure was the use of b/tsDMARDs. Multivariable logistic regression was used to assess the association between b/tsDMARDs treatment and hand joint deformity. *Results:* A total of 1083 RA patients with a mean age of 52.6 ± 12.4 years and a median disease duration of 5 (2,11) years were included. Hand joint deformity was present in 25.4% (275/1083) of patients. The top three deformed joint locations were PIP V (12.9%, 140/1083), PIP III (11.6%, 126/1083), and PIP IV (10.9%, 118/1083). The top three deformity types were ulnar deviation of MCP II-V (8.0%, 87/1083), boutonniere deformity of II-V fingers (6.8%, 74/1083), and swan neck deformity of II-V fingers (6.7%, 73/1083). In total, 17.4% (188/1083) of patients had received b/tsDMARDs. After 1:1 propensity score matching for age, sex, and disease duration, the prevalence of deformity was significantly lower in patients treated with conventional medicine (csDMARDs and/or GCs) add-on b/tsDMARDs compared to those treated with conventional medicine (27.1% vs. 61.7%, *p* < 0.001). Multivariable logistic regression analysis showed that b/tsDMARDs use was independently associated with a lower prevalence of hand joint deformity after adjusting for confounding factors (OR = 0.211, 95%CI: 0.129–0.345, *p* < 0.001). *Conclusions*: The use of b/tsDMARDs was independently associated with a lower prevalence of hand joint deformity in RA.

## 1. Introduction

Rheumatoid arthritis (RA) is a systemic autoimmune disease characterized by chronic symmetrical polyarthritis. The hallmark pathological features include synovial inflammatory hyperplasia and pannus formation, which progressively destroy articular cartilage, cause bone erosion, and ultimately lead to structural joint damage [1,2]. Hand deformities, such as ulnar deviation, swan neck, and boutonniere deformities, represent particularly disabling outcomes, which can severely impair hand function, grip strength, and quality of life [3,4]. Evidence suggests that irreversible joint damage affects 30% to 70% of patients, often due to delayed diagnosis, inadequate treatment response, or restricted access to advanced therapies [5,6]. Thus, early and effective intervention is essential to reduce the risk of deformity and preserve long-term joint function.

The treatment paradigm for RA has evolved significantly with the advent of biologic and targeted-synthetic disease-modifying antirheumatic drugs (b/tsDMARDs), which act on specific inflammatory pathways to suppress disease activity and inhibit structural damage [7,8,9]. While randomized controlled trials have consistently demonstrated their efficacy in slowing radiographic progression [10], real-world evidence regarding their effectiveness, particularly in Chinese populations, in preventing clinically evident hand joint deformities is limited. Most existing epidemiological studies on hand deformities derive from Western cohorts treated in the pre-b/tsDMARD era or focus on radiographic endpoints rather than clinically apparent deformity [11]. Thus, the relationship between contemporary treatment strategies and the actual prevalence of hand deformities in real-world settings is not well established.

To address this evidence gap, we conducted a cross-sectional analysis using data from a prospective real-world cohort of Chinese RA patients enrolled between 2019 and 2024. This study aimed to assess whether b/tsDMARDs therapy is associated with a lower prevalence of hand joint deformity. Our findings may inform clinical strategies aimed at preventing physical disability and improving long-term outcomes in RA.

## 2. Materials and Methods

### 2.1. Study Design and Participants

This study was derived from our ongoing real-world prospective RA cohort study conducted at the Department of Rheumatology and Immunology at Sun Yat-Sen Memorial Hospital, Sun Yat-Sen University. The diagnosis of RA was based on the 2010 American College of Rheumatology/European League Against Rheumatism classification criteria [12]. RA patients recruited from January 2019 to July 2024 who had undergone a complete clinical assessment, including hand joint deformity, were included in the present analysis [13]. Exclusion criteria included combinations of other autoimmune diseases (e.g., systemic lupus erythematosus, systemic sclerosis, dermatomyositis), malignant tumors, or conditions potentially affecting hand function, such as hemiplegia, upper limb fractures or surgeries within the past six months, and congenital or acquired hand joint deformities unrelated to RA (e.g., traumatic deformities, nerve or tendon injuries).

The study was performed in accordance with the ethical standards laid down in the 1964 Declaration of Helsinki. The Ethics Committee of Sun Yat-Sen Memorial Hospital approved this study (SYSEC-KY-KS-012). All patients signed informed consent forms.

### 2.2. Clinical Assessments

Demographic and clinical data were collected at baseline as our previous study reported [14,15], including age, sex, active smoking, body mass index (BMI), disease duration, disease activity, hand function, radiographic indicators, and previous medications. Treatment naïve was defined as no prior exposure to glucocorticoids (GCs), conventional synthetic disease-modifying antirheumatic drugs (csDMARDs), or b/tsDMARDs before enrollment.

Based on medication history, patients were categorized into two exposure groups for the primary analysis: (1) those treated with conventional medicine alone (csDMARDs and/or GCs), and (2) those receiving conventional medicine (csDMARDs and/or GCs) add-on b/tsDMARDs (including tumor necrosis factor inhibitors [TNFi], interleukin-6 inhibitors [IL-6i], or Janus kinase inhibitors [JAKi]).

Disease activity indicators included tender and swollen joint counts (28TJC, 28SJC), patient/physician global disease activity assessments (PtGA, PrGA), and pain visual analog scale (VAS). Laboratory measures included rheumatoid factor (RF), anti-cyclic citrullinated peptide antibody (ACPA), erythrocyte sedimentation rate (ESR, normal value < 15 mm/h in men and <20 mm/h in women), and C-reactive protein (CRP, normal value < 5 mg/L). The normal ESR/CRP subgroup was defined as having both ESR and CRP within normal values, while the elevated ESR/CRP subgroup was defined as having an elevation in either ESR or CRP. Active RA was defined as the clinical disease activity index (CDAI) > 2.8.

Hand grip strength (kg) was measured using a dynamometer (Smedley, Shanghai, China). Two measurements were taken for each hand. The highest reading from the stronger hand was used for analysis, as it is less susceptible to the number of attempts than the mean value. Decreased grip strength was defined as less than 28.0 kg for males and 18.0 kg for females. Self-reported hand disability was evaluated using the validated Chinese version of the Stanford Health Assessment Questionnaire disability index (HAQ-DI) [16]. The HAQ-DI assesses hand disability using eight items related to dressing, eating, and grip. Each item is scored 0 to 3 (0 = no difficulty, 1 = some difficulty, 2 = much difficulty, 3 = unable to do, Appendix S1). The scores were averaged to yield a HAQ-DI hand disability score ranging from 0 to 3. A score > 0 was defined as indicating the presence of self-reported HAQ-DI hand disability [16,17].

Conventional radiographic assessments of the bilateral hands and wrists were conducted using the Sharp/van der Heijde modified Sharp score, which includes the modified total Sharp score (mTSS) along with subscores for joint erosion (JE) and joint space narrowing (JSN) [18]. Subjects with mTSS > 10 were considered to have radiographic joint damage (RJD) [19].

### 2.3. Assessment of Hand Joint Deformity

All deformity assessments were performed by experienced and well-trained rheumatologists who receive regular training at least twice a year, which ensures internal consistency. The mean intra-class correlation coefficient (ICC) for inter-rater reliability was 0.972 during the training. The location, type, and number of hand joint deformities were assessed via physical examination in 28 joints of both hands, including the metacarpophalangeal (MCP) joints I-V, proximal interphalangeal (PIP) joints I-V, and distal interphalangeal (DIP) joints II-V. Hand joint deformities were classified into the following types: subluxation (ulnar or radial deviation), hyperflexion, hyperextension, boutonniere deformity, swan neck deformity, and Z-deformity of the thumb [20,21]. The boutonniere (French for button hole) deformity is a result of an injury to the finger extensor mechanism that causes characteristic flexion of the PIP joint and hyperextension of the DIP joint [22]. The swan neck deformity is characterized by PIP joint hyperextension and flexion of the DIP joint [23]. The Z-deformity of the thumb is characterized by flexion at the MCP I joint and hyperextension at the PIP I joint [24,25]. The presence of at least one of the above-mentioned deformities in either hand was defined as hand joint deformity.

### 2.4. Statistical Analysis

All statistical analyses were performed using R version 4.5.1 (R Foundation for Statistical Computing, Vienna, Austria). Data are presented as mean ± Standard Deviation (SD) for normally distributed variables, median (25th, 75th percentiles) for non-normally distributed variables, and n (%) for categorical variables. Comparisons of continuous variables between patients with and without hand joint deformity were performed using the independent samples *t*-test or the Mann–Whitney U test, as appropriate. Categorical variables were compared using Chi-squared tests or Fisher’s exact tests. Patients in the conventional medicine (csDMARDs and/or GCs) and conventional medicine (csDMARDs and/or GCs) add-on b/tsDMARDs groups were matched 1:1 using propensity scores derived from age, sex, and disease duration. Univariable and multivariable logistic regression models were used to assess the association between the use of b/tsDMARDs and hand joint deformity in RA patients. Model 1 was adjusted for age, sex, disease duration, and active smoking. Model 2 was further adjusted for positive RF and positive ACPA. Model 3 was additionally adjusted for morning stiffness time, pain VAS, ESR, and CRP. Model 4 was also adjusted for the CDAI and mTSS. Subgroup analyses were conducted based on the use of MTX and b/tsDMARDs, as well as the specific types of b/tsDMARDs (TNFi, IL-6i, and JAKi). Pairwise comparisons among multiple groups were corrected using the Bonferroni correction. An interaction analysis between the disease duration and b/tsDMARDs use was performed. A two-sided *p* value < 0.05 was considered statistically significant.

## 3. Results

### 3.1. Demographic and Clinical Characteristics of Patients with RA

**Figure 1:**
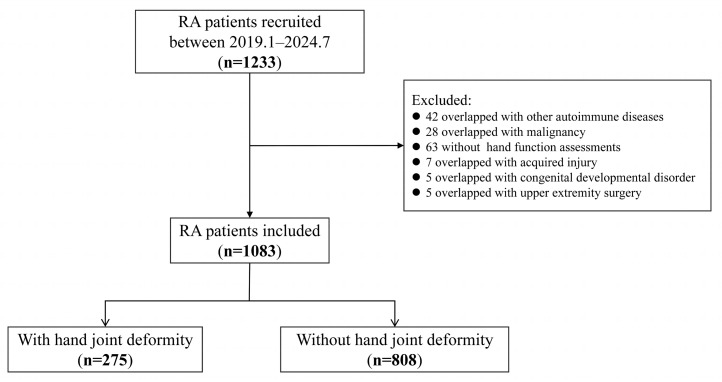
The flowchart of patient selection.

A total of 1233 RA patients were recruited from January 2019 to July 2024. After excluding 42 patients with other autoimmune diseases, 28 patients with malignancy, 63 patients with incomplete assessment of hand function, 7 patients with acquired hand injury, 5 patients with congenital disorders of fingers, and 5 patients who had a history of upper extremity surgery, a total of 1083 patients were included in the final analysis (Figure 1). Among them, 82.9% (898/1083) of patients were female, with a mean age of 52.6 ± 12.4 years and a median disease duration of 5 (2,11) years. According to CDAI, 75.3% (816/1083) of patients had active RA. HAQ hand disability was present in 39.2% (424/1083) of patients, and 72.1% (781/1083) had decreased grip strength. Radiographic joint damage was observed in 52.1% (564/1083) of patients. Overall, 15.4% (167/1083) of patients were treatment naïve at enrollment. Methotrexate was the most commonly used csDMARDs (68.9%, 746/1083), followed by leflunomide (38.8%, 420/1083), hydroxychloroquine (37.8%, 409/1083), and sulfasalazine (4.9%, 53/1083). There were 17.4% (188/1083) patients with b/tsDMARDs, 8.6% (93/1083) with JAK inhibitors, 4.9% (53/1083) with TNF inhibitors, and 3.9% (42/1083) with IL-6 inhibitors (Table 1).

### 3.2. Characteristics of Hand Joint Deformity in Patients with RA

**Figure 2:**
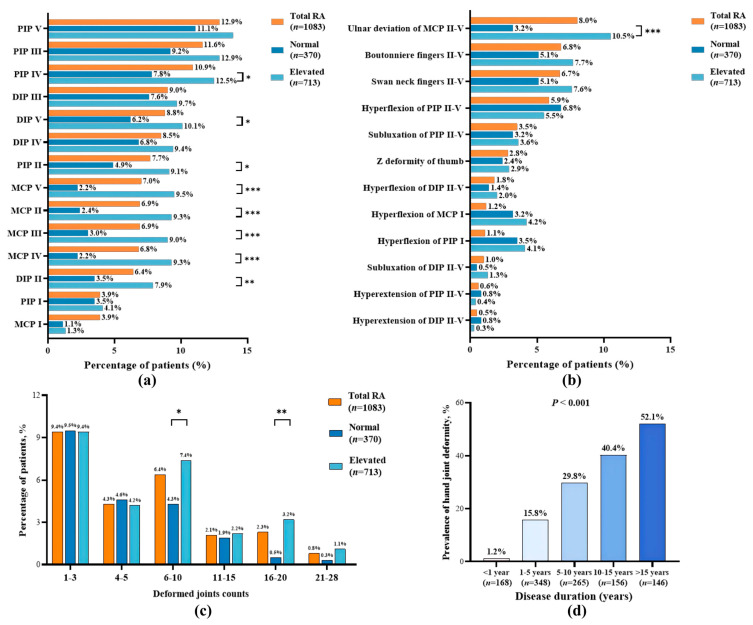
Characteristics of hand joint deformity in patients with RA. (**a**) Locations of hand joint deformities; (**b**) Types of hand joint deformities; (**c**) Percentage of patients according to the number of deformed joint counts; (**d**) Prevalence of hand joint deformity stratified by disease duration. MCP, Metacarpophalangeal joint; PIP, Proximal Interphalangeal Joint; DIP, Distal Interphalangeal joint; Normal, defined as having both ESR and CRP within normal values; Elevated, defined as having elevated ESR or CRP. * *p* < 0.05, ** *p* < 0.01, *** *p* < 0.001 for comparisons between groups.

Hand joint deformity was present in 25.4% (275/1083) of RA patients. Among them, the median number of deformed joints was 5 (2, 9), and the maximum number of deformed joints was 28. The top 3 deformed joint locations were PIP V (12.9%, 140/1083), PIP III (11.6%, 126/1083), and PIP IV (10.9%, 118/1083) joints. The most common deformity types were ulnar deviation of MCP II-V (8.0%, 87/1083), boutonniere deformity of fingers II-V (6.8%, 74/1083), and swan neck deformity of fingers II-V (6.7%, 73/1083) (Figure 2a,b). The number of deformed joints counted per patient was categorized into six groups: 1–3, 4–5, 6–10, 11–15, 16–20, and 21–28. Among the RA patients, 9.4% (102/1083) had deformity counts in the 1–3 range, 6.4% (69/1083) fell into the 6–10 range. Deformity prevalence increased with disease duration, rising notably after 5 years (Figure 2c,d).

Patients were divided into two groups based on ESR and CRP levels: the normal ESR and CRP group (34.2%, 370/1083) and the elevated ESR/CRP group (65.8%, 713/1083). Compared with the normal group, the elevated ESR/CRP group was older (mean ± SD: 54.3 ± 11.8 years vs. 49.3 ± 12.9 years), had a lower proportion of females (80.9% vs. 86.8%), a higher proportion of positive RF (72.2% vs. 57.6%), a higher CDAI score (median 12 vs. 5), a lower proportion of CDAI remission (17.0% vs. 39.5%), and a higher proportion of decreased grip strength (79.2% vs. 58.4%), HAQ hand disability (46.4% vs. 25.1%), and RJD (56.2% vs. 44.1%; all *p* < 0.05, Table S1). The elevated group had a higher overall prevalence of hand joint deformity (27.6%, 197/713) than the normal group (21.1%, 78/370; *p* < 0.05). Deformities at specific joints (MCP II, III, IV, V; PIP II, IV; DIP II, V) and ulnar deviation of MCP II-V were more common in the elevated group (all *p* < 0.05, Figure 2a,b). The elevated group also had a higher proportion of patients with 6–10 or 16–20 deformed hand joints (7.4% vs. 4.3%; 3.2% vs. 0.5%, respectively; both *p* < 0.05, Figure 2c).

### 3.3. Clinical Characteristics of RA Patients with and Without Hand Joint Deformity

Patients with hand joint deformity were significantly older (54.4 vs. 52.0 years), had a longer disease duration (median 10 vs. 4 years), higher CDAI (median 11 vs. 8), higher proportion of decreased grip strength (84.0% vs. 68.1%), higher proportion of HAQ hand disability (45.1% vs. 37.1%), higher mTSS (median 29 vs. 11), and higher proportion of glucocorticoid treatment than those without deformity (57.5% vs. 45.2%, all *p* < 0.01, Table 1).

In the subgroup of patients with elevated ESR or CRP, patients with deformity had higher PtGA (median 4 vs. 3), PrGA (median 3 vs. 2), CDAI (median 14 vs. 11), and mTSS (median 32 vs. 13) than patients without deformity (all *p* < 0.05, Table S2). In the subgroup of patients with elevated ESR or CRP, after matching for disease duration, those with deformity had a higher CDAI (median 14 vs. 10) and a higher mTSS (median 32 vs. 23, all *p* < 0.05). Similarly, in the subgroup with normal ESR and CRP, patients with deformity still had more severe radiographic joint damage, indicated by higher mTSS (median 25 vs. 10) and a higher prevalence of RJD (66.7% vs. 44.7%) (both *p* < 0.05, Table S3).

### 3.4. Association Between b/tsDMARDs and Hand Joint Deformity

**Figure 3:**
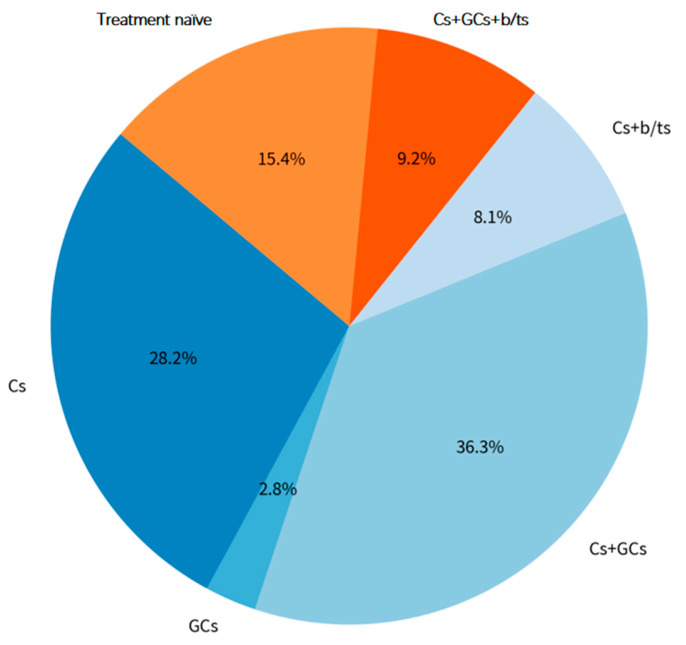
Treatment history and drug exposure in patients with RA. DMARDs, disease-modifying anti-rheumatic drugs; Cs, csDMARDs, conventional synthetic DMARDs; GCs, glucocorticoids; b/ts, biological or targeted-synthetic DMARDs.

Among the enrolled patients, the proportions with different treatment histories were as follows: 15.4% (167/1083) were treatment naïve; 36.3% (393/1083) had received combined csDMARDs and GCs therapy; 28.2% (305/1083) had received csDMARDs monotherapy; 9.2% (100/1083) had received the triple-drug combination of csDMARDs, GCs, and b/tsDMARDs; 8.1% (88/1083) had received the combination of csDMARDs and b/tsDMARDs; and 2.8% (30/1083) had received GCs monotherapy (Figure 3).

Patients were categorized into two groups to assess the association between b/tsDMARDs treatment and the prevalence of hand joint deformity: those receiving conventional medicine (csDMARDs and/or GCs) (*n* = 728) and those receiving conventional medicine (csDMARDs and/or GCs) add-on b/tsDMARDs (*n* = 188). After propensity score matching for age, sex, and disease duration (188 patients in each group), a striking disparity in the prevalence of hand joint deformity was observed. Patients receiving conventional medicine add-on b/tsDMARDs had a significantly lower overall prevalence of deformity compared to those on conventional medicine (csDMARDs and/or GCs) (27.1% vs. 61.7%, *p* < 0.001). This association was consistent across most specific deformity types. Specifically, the conventional medicine add-on b/tsDMARDs group exhibited markedly reduced prevalence of boutonniere deformity (6.9% vs. 14.4%), swan neck deformity (6.9% vs. 16.0%), ulnar deviation of MCP joints (8.0% vs. 19.1%), PIP joints hyperflexion (3.7% vs. 17.6%) and DIP joints hyperextension (0% vs. 2.1%, all *p* < 0.05, Table 2).

We further performed subgroup analyses based on the use of different b/tsDMARDs among the matched RA patients: conventional medicine, and conventional medicine with add-on TNFi, IL-6i, and JAKi. Specifically, the prevalence of hand joint deformity was lower in all b/tsDMARDs add-on groups (TNFi: 28.8% [15/52]; IL-6i: 38.1% [16/42]; JAKi: 22.0% [20/91]) compared with the conventional medicine alone group (61.7% [116/188]). However, pairwise comparisons among the three b/tsDMARDs subgroups revealed that the prevalence of hyperflexion of PIP II-V was significantly lower in the JAKi add-on group (0%, 0/91) than in the TNFi (5.8%, 3/52) and IL-6i (9.5%, 4/42) add-on group (*p* < 0.0083, Bonferroni correction, Table S4). We also compared the prevalence of deformity across groups defined by MTX and b/tsDMARDs use. The prevalence of deformity was significantly lower in both the non-MTX + b/tsDMARDs group (28.6%, 10/35) and the MTX + b/tsDMARDs group (26.8%, 41/153) compared with the non-MTX + non-b/tsDMARDs group (66.7%, 28/42) (both *p* < 0.0083, Bonferroni correction; Table S5).

In the multivariable logistic regression analysis, b/tsDMARDs use was independently associated with a lower prevalence of hand joint deformity (Table 3). This association persisted across sequentially adjusted models. Even after comprehensive adjustment for active smoking, serological status, inflammatory markers, disease activity (CDAI), and mTSS, the association remained highly significant (adjusted OR = 0.211, 95% CI: 0.129–0.345, *p* < 0.001). Analysis of specific b/tsDMARDs classes revealed that, compared with conventional medicine alone, each was independently associated with a lower prevalence of deformity after full adjustment (TNFi: OR = 0.216, 95%CI: 0.096–0.487; IL-6i: OR = 0.338, 95%CI: 0.154–0.742; JAKi: OR = 0.162, 95%CI: 0.084–0.311; all *p* < 0.05, Table 3). We also analyzed the associations of MTX and b/tsDMARDs subgroups with hand joint deformity in patients with RA. Compared with patients treated with non-MTX + non-b/tsDMARDs, those with MTX + b/tsDMARDs had a significant independent association with a lower prevalence of hand joint deformity after adjusting all confounders (OR = 0.180, 95%CI: 0.079–0.412, *p* < 0.001, Table S6).

Furthermore, we conducted stratified analysis based on the disease duration (<5 years vs. ≥5 years). The results showed that b/tsDMARDs use remained significantly associated with a lower prevalence of deformity in both disease duration groups (<5 years and ≥5 years). However, in the subgroup analysis by b/tsDMARDs types, this significant association was observed only in patients with a disease duration ≥ 5 years (TNFi: OR = 0.150, 95%CI: 0.047–0.479; IL-6i: OR = 0.174, 95%CI: 0.059–0.510; JAKi: OR = 0.080, 95%CI: 0.033–0.194; all *p* < 0.05, Table 4). The interaction analysis indicated a significant interaction between b/tsDMARDs use and disease duration (OR = 0.929, 95%CI: 0.890–0.969, *p* = 0.001). Specifically, significant interactions with disease duration were also observed for IL-6i (OR = 0.915, 95%CI: 0.852–0.981) and JAKi (OR = 0.884, 95%CI: 0.828–0.943, both *p* < 0.05, Table 4).

## 4. Discussion

This cross-sectional study revealed that 25.4% of RA patients recruited from 2019 to 2024 had hand joint deformity. Patients with hand joint deformity had higher disease activity, greater functional limitation, decreased grip strength, and more severe radiographic joint damage than those without. The use of b/tsDMARDs was significantly associated with a lower prevalence of hand joint deformity (OR = 0.211) after adjusting for confounding factors. There was a significant interaction between b/tsDMARDs use and disease duration on the prevalence of hand joint deformity (OR = 0.929), indicating that the association of b/tsDMARDs increased with the prolongation of the disease duration.

Hand joint deformity is a major complication of RA, with reported prevalence ranging from 40% to 90% [3,26,27]. The most prevalent deformity is the MCP joint ulnar deviation, followed by the swan neck deformity or boutonniere deformity [28]. A cross-sectional analysis of 122 RA patients showed that 26.2% had ulnar deviation deformity, 14.8% had boutonniere deformity, and 13.9% had swan-neck deformity [29]. Another small-sample study (n = 33) reported a higher prevalence, with 45.5% of patients exhibiting more than 1 deformity type [26], highlighting the variability across studies. It has been confirmed that disease duration is strongly correlated with deformity severity. A prospective cohort of 100 early RA patients (mean disease duration 11 months) demonstrated incident deformities in 31% after 2 years [11]. Another study reported that 45% of RA patients with > 5 years of disease duration had hand joint deformity [30]. In our large-scale study of 1083 RA patients, the prevalence of hand joint deformity was 25.4% (275/1083). Ulnar deviation was present in 8.0% (87/1083) of patients, followed by boutonniere deformity (6.8%, 74/1083) and swan neck deformity (6.7%, 73/1083). Consistent with prior evidence, we found that deformity burden is positively correlated with disease duration. The risk of hand joint deformity rises gradually over time, with a notable acceleration after approximately 5–10 years of disease duration. These findings collectively confirm a still high prevalence of RA-associated hand joint deformity in the recent five years.

Rheumatoid arthritis significantly impacts quality of life, as the course of joint destruction compromises physical function, daily activities, and work capacity. A study of 183 RA patients from Sweden showed that those with deformity had higher HAQ-DI during the first 10 years [31]. Similarly, a study of 779 patients from the United States reported a strong direct association between hand joint deformity and functional limitation (standardized regression coefficients = −0.564, *p* < 0.001) [32]. Our study in a Chinese cohort corroborates these findings, demonstrating a higher prevalence of HAQ hand disability (45.1% vs. 37.1%) and decreased grip strength (84.0% vs. 68.1%) in patients with deformities than in those without. Given that hand joint deformity is a serious consequence of RA and a key factor impairing daily function, yet remains understudied in contemporary research, our findings provide robust evidence of its association with functional limitation. While the strong cross-sectional association we observed between b/tsDMARDs use and a lower prevalence of deformity aligns with the known role of these drugs in inhibiting radiographic progression, the observational design of our study precludes causal inference. Therefore, these results suggest that clinical management strategies should prioritize the monitoring and early intervention for hand joint deformity to preserve long-term function and quality of life.

Previous studies, including one by Johnsson et al., have demonstrated that hand joint deformity is significantly associated with higher disease activity [33]. Our findings are consistent with these reports, showing higher disease activity in patients with hand joint deformity, irrespective of their ESR and CRP levels. In our study, no significant difference was observed in the prevalence of positive RF or ACPA between RA patients with and without hand joint deformities. This may be because the majority of patients were already receiving DMARDs, which could have attenuated the association between positive RF or ACPA and joint deformity by inhibiting inflammation and bone destruction. We also found that deformity was associated with an approximately 1.8-fold higher prevalence of radiographic joint damage (RJD). As a primarily non-inflammatory factor, deformity generates abnormal biomechanical stress that promotes radiographic joint damage through structural abnormalities, biomechanical imbalance, and abnormal joint loading. Deformed joints can increase local cartilage pressure, directly damage the cartilage matrix, and thereby accelerate cartilage loss [34]. Notably, RJD correlates more strongly with deformity severity than with CRP/ESR in established RA [35]. Furthermore, deformity also reduces joint mobility, induces muscle atrophy, and diminishes joint protection, which further drives RJD [36]. A study showed that patients with early deformities (≤1 year) had a 2.1-fold higher risk of severe Larsen scores at 5 years [33]. Therefore, early identification and management of hand joint deformities necessitate an integrated multidisciplinary approach, which is essential for improving long-term outcomes in rheumatoid arthritis.

The observed association between b/tsDMARDs use and a lower prevalence of deformity may be explained by the superior and targeted suppression of synovitis afforded by b/tsDMARDs. In our study, the use of TNFi IL-6i and JAKi was associated with a lower prevalence of deformity in patients with a disease duration of ≥5 years. There was a significant interaction between b/tsDMARDs use (especially IL-6i and JAKi) and disease duration, with lower deformity prevalence. These agents act on key inflammatory pathways such as TNF-α, IL-6, and JAK, thereby reducing synovitis and slowing bone erosion, processes central to the pathogenesis of joint deformity [2,37,38,39]. This mechanism aligns with the treat-to-target strategy endorsed by the 2019 EULAR recommendations, which emphasize that the primary goal of DMARD therapy is to prevent or inhibit structural damage [40]. Achieving sustained remission through this strategy is paramount for preventing long-term disability, especially for patients with a disease duration of more than 5 years.

Our study has several limitations. First, the cross-sectional nature of the study design precludes the determination of temporal sequence and causal inference. Specifically, we cannot rule out reverse causation; the absence of severe deformity might have influenced the decision to initiate b/tsDMARDs, or established deformities might have prompted more aggressive therapy. Therefore, future prospective longitudinal studies are essential to delineate temporal relationships and confirm the association observed in this study. Second, the assessment of deformities was based on physical examination without standardized quantitative measurements, such as joint range of motion or ulnar deviation angles. Future prospective studies will incorporate quantitative functional assessment to improve measurement quality. Third, this was an observational study, and treatment was not randomly assigned. The decision to use b/tsDMARDs was based on real-world clinical judgment, which may introduce confounding by indication, despite our statistical adjustments. Future randomized controlled trials with larger cohorts are needed to explore potential differential effects between individual agents with distinct mechanisms of action. Finally, this was a single-center study conducted in a Chinese population. The generalizability of our findings to other geographic regions or ethnic groups may be limited and warrants external validation in independent, multi-center cohorts.

## 5. Conclusions

In this contemporary Chinese cohort, hand joint deformity was present in one-fourth of patients with RA. We found an association between the use of b/tsDMARDs and a lower prevalence of hand joint deformity in RA, and this association increased with the prolongation of the disease duration. Future prospective studies are needed to confirm these associations and explore whether b/tsDMARDs may help prevent structural damage in patients with RA. Such studies should focus on optimizing holistic care protocols, integrating pharmacological prevention with physical therapy and surgical intervention, to manage deformity and improve outcomes for affected patients.

## Figures and Tables

**Table 1:** Comparison of demographic and clinical characteristics between RA patients with and without hand joint deformity.

Characteristics	All Patients (*n* = 1083)	Without Deformity (*n* = 808)	With Deformity (*n* = 275)	*p*
Age, years	52.6 ± 12.4	52.0 ± 2.6	54.4 ± 11.6	0.006
Female, *n* (%)	898 (82.9)	661 (81.8)	237 (86.2)	0.096
Active smoking, *n* (%)	142 (13.1)	106 (13.1)	36 (13.1)	0.991
Disease duration, years	5 (2, 11)	4 (1, 8)	10 (6, 16)	<0.001
Positive RF, *n* (%)	728 (67.2)	535 (66.2)	193 (70.2)	0.226
Positive ACPA, *n* (%)	717 (66.2)	526 (65.1)	191 (69.5)	0.187
Core disease activity indicators				
Morning stiffness time, min	0 (0, 8)	0 (0, 10)	0 (0, 5)	0.143
28TJC	2 (0, 6)	2 (0, 6)	2 (0, 7)	0.073
28SJC	1 (0, 3)	1 (0, 3)	1 (0, 4)	0.074
PtGA, cm	2 (1, 5)	2 (0, 4)	3 (2, 5)	<0.001
PrGA, cm	2 (1, 4)	2 (0, 4)	3 (1, 5)	<0.001
Pain VAS, cm	2 (1, 4)	2 (0, 4)	2 (1, 4)	0.010
ESR (mm/h)	25 (14, 50)	25 (13, 47)	29 (16, 52)	0.040
CRP (mg/L)	4 (3, 11)	4 (3, 11)	4 (3, 11)	0.634
CDAI	9 (3, 18)	8 (2, 17)	11 (5, 20)	<0.001
CDAI remission, *n* (%)	267 (24.7)	223 (27.6)	44 (16.0)	<0.001
Low disease activity, *n* (%)	292 (27.0)	215 (26.6)	77 (28.0)	
Middle disease activity, *n* (%)	315 (29.1)	225 (27.8)	90 (32.7)	
High disease activity, *n* (%)	209 (19.3)	145 (17.9)	64 (23.3)	
Hand function indicators				
Decreased grip strength, *n* (%)	781 (72.1)	550 (68.1)	231 (84.0)	<0.001
HAQ hand score	0.00 (0.00, 0.25)	0.00 (0.00, 0.25)	0.00 (0.00, 0.38)	0.008
HAQ hand disability, *n* (%)	424 (39.2)	300 (37.1)	124 (45.1)	0.019
Radiographic assessment				
mTSS	15 (4, 9)	11 (3, 29)	29 (11, 62)	<0.001
JSN	4 (0, 14)	3 (0, 10)	11 (4, 32)	<0.001
JE	9 (3, 22)	8 (2, 18)	15 (5, 34)	<0.001
RJD, *n* (%)	564 (52.1)	367 (45.4)	197 (71.6)	<0.001
Previous medications				
Treatment naïve, *n* (%)	167 (15.4)	151 (18.7)	16 (5.8)	<0.001
Glucocorticoids, *n* (%)	523 (48.3)	365 (45.2)	158 (57.5)	<0.001
csDMARDs, *n* (%)	878 (81.1)	638 (78.2)	246 (89.5)	<0.001
methotrexate, *n* (%)	746 (68.9)	541 (67.0)	205 (74.5)	0.019
sulfasalazine, *n* (%)	53 (4.9)	34 (4.2)	19 (6.9)	0.073
hydroxychloroquine, *n* (%)	409 (37.8)	291 (36.0)	118 (42.9)	0.042
leflunomide, *n* (%)	420 (38.8)	295 (36.5)	125 (45.5)	0.009
b/tsDMARDs, *n* (%)	188 (17.4)	137 (17.1)	51 (18.5)	0.548
IL-6i, *n* (%)	42 (3.9)	26 (3.2)	16 (5.8)	0.054
TNFi, *n* (%)	53 (4.9)	38 (4.7)	15 (5.5)	0.618
JAKi, *n* (%)	93 (8.6)	73 (9.0)	20 (7.3)	0.368

RF, rheumatoid factor; ACPA, anti-cyclic citrullinated peptide antibody; 28TJC, 28-joint tender joint counts; 28SJC, 28-joint swollen joint counts; PtGA, patient global assessment of disease activity; PrGA, provider global assessment of disease activity; Pain VAS, pain visual analog scale; ESR, erythrocyte sedimentation rate; CRP, C-reactive protein; CDAI, Clinical Disease Activity Index; HAQ, Health Assessment Questionnaire; mTSS, modified total Sharp score; JSN, joint space narrowing; JE, joint erosion; RJD, radiographic joint damage; DMARDs, disease-modifying anti-rheumatic drugs; treatment naïve, having no previous glucocorticoids or DMARDs therapy before enrollment; csDMARDs, conventional synthetic DMARDs; bDMARDs, biological DMARDs; tsDMARDs, targeted-synthetic DMARDs; TNFi, tumor necrosis factor inhibitors; IL-6i, interleukin-6 inhibitors; JAKi, Janus kinase inhibitors.

**Table 2:** Comparison of patient characteristics and hand joint deformity by previous treatment.

Characteristics	Before Matching			After Matching		
	Conventional Medicine (*n* = 728)	Conventional Medicine + b/ts (*n* = 188)	*p*	Conventional Medicine (*n* = 188)	Conventional Medicine + b/ts (*n* = 188)	*p*
Age, years	53.1 ± 12.2	49.6 ± 12.5	0.001	49.9 ± 12.8	49.6 ± 12.5	0.810 ^a^
Female, *n* (%)	606 (83.2)	161 (85.6)	0.427	160 (85.1)	161 (85.6)	0.884 ^b^
Disease duration, years	6 (2, 11)	5 (2, 11)	0.732	7 (3, 12)	5 (2, 11)	0.331 ^c^
Positive RF, *n* (%)	476 (65.4)	134 (71.3)	0.127	122 (64.9)	134 (71.3)	0.184
Positive ACPA, *n* (%)	475 (65.2)	137 (72.9)	0.048	133 (70.7)	137 (72.9)	0.647
ESR (mm/h)	25 (14, 46)	25 (11, 45)	0.643	26 (16, 50)	25 (11, 45)	0.239
CRP (mg/L)	3 (3, 10)	3 (3, 9)	0.618	3 (3, 10)	3 (3, 9)	0.947
CDAI	8 (2, 16)	8 (3, 14)	0.421	8 (3, 16)	8 (3, 14)	0.923
CDAI remission, *n* (%)	216 (29.7)	40 (21.3)	0.022	45 (23.9)	40 (21.3)	0.538
Decreased grip strength, *n* (%)	508 (69.8)	138 (73.4)	0.331	130 (69.1)	138 (73.4)	0.362
HAQ hand score	0.00 (0.00, 0.13)	0.00 (0.00, 0.13)	0.896	0.00 (0.00, 0.13)	0.00 (0.00, 0.13)	0.732
HAQ hand disability, *n* (%)	252 (34.6)	66 (35.1)	0.900	68 (36.2)	66 (35.1)	0.829
RJD, *n* (%)	372 (51.1)	108 (57.4)	0.037	108 (57.4)	108 (57.4)	0.438
Hand joint deformity, *n*(%)	208 (28.6)	51 (27.1)	0.695	116 (61.7)	51 (27.1)	<0.001
Type of deformity						
Boutonniere fingers II-V, *n* (%)	57 (7.8)	13 (6.9)	0.674	27 (14.4)	13 (6.9)	0.019
Swan neck fingers II-V, *n* (%)	56 (7.7)	13 (6.9)	0.719	30 (16.0)	13 (6.9)	0.006
Z deformity of thumb, *n* (%)	23 (3.2)	6 (3.2)	0.982	9 (4.8)	6 (3.2)	0.429
Ulnar deviation of MCP II-V, *n* (%)	63 (8.7)	15 (8.0)	0.767	36 (19.1)	15 (8.0)	0.002
Hyperflexion of MCP I, *n* (%)	11 (1.5)	2 (1.1)	0.633	5 (2.7)	2 (1.1)	0.245
Hyperflexion of PIP I, *n* (%)	8 (1.1)	3 (1.6)	0.590	3 (1.6)	3 (1.6)	-
Hyperflexion of PIP II-V, *n* (%)	54 (7.4)	7 (3.7)	0.070	33 (17.6)	7 (3.7)	<0.001
Hyperextension of PIP II-V, *n* (%)	4 (0.5)	1 (0.5)	0.977	3 (1.6)	1 (0.5)	0.304
Subluxation of PIP II-V, *n* (%)	27 (3.7)	9 (4.8)	0.498	18 (9.6)	9 (4.8)	0.072
Hyperflexion of DIP II-V, *n* (%)	16 (2.2)	3 (1.6)	0.594	7 (3.7)	3 (1.6)	0.200
Hyperextension of DIP II-V, *n* (%)	5 (0.7)	0 (0)	0.129	4 (2.1)	0 (0)	0.018
Subluxation of DIP II-V, *n* (%)	9 (1.2)	2 (1.1)	0.844	5 (2.7)	2 (1.1)	0.245

^a^ Standardized mean difference (SMD) = 0.025; ^b^ SMD = 0.015; ^c^ SMD = 0.08. DMARDs, disease-modifying anti-rheumatic drugs; Conventional medicine, conventional DMARDs and/or GCs; b/ts, biological or targeted-synthetic disease-modifying antirheumatic drugs; RF, rheumatoid factor; ACPA, anti-cyclic citrullinated peptide antibody; ESR, erythrocyte sedimentation rate; CRP, C-reactive protein; CDAI, Clinical Disease Activity Index; HAQ, Health Assessment Questionnaire; RJD, radiographic joint damage; MCP, metacarpophalangeal joint; PIP, proximal interphalangeal joint; DIP, distal interphalangeal joint.

**Table 3:** Association between b/tsDMARDs use and hand joint deformity in patients with RA.

Model	b/tsDMARDs		TNFi		IL-6i		JAKi	
	OR (95% CI)	*p*	OR (95% CI)	*p*	OR (95% CI)	*p*	OR (95% CI)	*p*
Crude Model	0.231 (0.149–0.357)	<0.001	0.252 (0.129–0.491)	<0.001	0.382 (0.192–0.761)	0.006	0.175 (0.098–0.311)	<0.001
Model 1	0.232 (0.150–0.359)	<0.001	0.264 (0.128–0.545)	<0.001	0.330 (0.157–0.693)	0.003	0.149 (0.080–0.277)	<0.001
Model 2	0.229 (0.147–0.355)	<0.001	0.260 (0.126–0.536)	<0.001	0.323 (0.153–0.684)	0.003	0.149 (0.080–0.278)	<0.001
Model 3	0.224 (0.143–0.349)	<0.001	0.236 (0.111–0.500)	<0.001	0.339 (0.159–0.725)	0.005	0.145 (0.077–0.273)	<0.001
Model 4	0.211 (0.129–0.345)	<0.001	0.216 (0.096–0.487)	<0.001	0.338 (0.154–0.742)	0.007	0.162 (0.084–0.311)	<0.001

Model 1: Adjusted for Age, Sex, Disease duration, Active smoking. Model 2: Model 1 + RF and ACPA. Model 3: Model 2 + Morning stiffness, Pain VAS, ESR, CRP. Model 4: Model 3 + CDAI + mTSS. DMARDs, disease-modifying anti-rheumatic drugs; b/tsDMARDS, biological or targeted-synthetic DMARDs; OR, Odds Ratio; CI, Confidence Interval; RF, rheumatoid factor; ACPA, anti-cyclic citrullinated peptide antibody; Pain VAS, pain visual analog scale; ESR, erythrocyte sedimentation rate; CRP, C-reactive protein; CDAI, Clinical Disease Activity Index; mTSS, modified total Sharp score; TNFi, tumor necrosis factor inhibitors; IL-6i, interleukin-6 inhibitors; JAKi, Janus kinase inhibitors.

**Table 4:** Interaction analyses of the associations of disease duration and b/tsDMARDs with hand joint deformity in patients with RA.

Subgroups	Multivariable Logistic Regression *	
	OR (95% CI)	*p*
b/tsDMARDs		
<5 years	0.409 (0.182–0.920)	0.031
≥5 years	0.110 (0.054–0.222)	<0.001
Interaction (b/tsDMARDs × disease duration)	0.929 (0.890–0.969)	0.001
TNFi		
<5 years	0.305 (0.078–1.194)	0.088
≥5 years	0.150 (0.047–0.479)	0.001
Interaction (TNFi × disease duration)	0.934 (0.872–1.001)	0.054
IL-6i		
<5 years	0.773 (0.199–2.995)	0.709
≥5 years	0.174 (0.059–0.510)	0.001
Interaction (IL-6i × disease duration)	0.915 (0.852–0.981)	0.013
JAKi		
<5 years	0.319 (0.100–1.024)	0.055
≥5 years	0.080 (0.033–0.194)	<0.001
Interaction (JAKi × disease duration)	0.884 (0.828–0.943)	<0.001

* Adjusted for age, sex, active smoking, positive RF and ACPA, morning stiffness, Pain VAS, ESR, CRP, CDAI, mTSS. b/tsDMARDS, biological or targeted-synthetic DMARDs; OR, Odds Ratio; CI, Confidence Interval; TNFi, tumor necrosis factor inhibitors; IL-6i, interleukin-6 inhibitors; JAKi, Janus kinase inhibitors.

## Data Availability

The original contributions presented in this study are included in the article/Supplementary Materials. Further inquiries can be directed to the corresponding authors.

## Supplementary Materials

The following supporting information can be downloaded at: https://www.mdpi.com/article/10.3390/medicina62020241/s1, Appendix S1. Questions related to hand function on the Stanford health assessment questionnaire (HAQ); Table S1: Comparison of demographic, clinical, and hand joint deformity characteristics between RA patients in normal and elevated ESR/CRP groups; Table S2: Comparison of clinical characteristics between RA patients with and without hand joint deformity, stratified by ESR/CRP status, before propensity score matching disease duration; Table S3: Comparison of clinical characteristics between RA patients with and without hand joint deformity, stratified by ESR/CRP status, after propensity score matching disease duration. Supplemental Table S4. Comparison of hand joint deformity characteristics by use of b/tsDMARDs in matched RA patients. Supplemental Table S5. Comparison of patient characteristics and hand joint deformity by use of MTX and b/tsDMARDs in matched RA patients. Supplemental Table S6. Association between subgroups of MTX and b/tsDMARDs with hand joint deformity in patients with RA.

## Abbreviations

The following abbreviations are used in this manuscript:

28TJC	28-joint tender joint count
28SJC	28-joint swollen joint count
ACPA	Anti-cyclic citrullinated peptide antibody
bDMARDs	Biological disease-modifying anti-rheumatic drugs
BMI	Body mass index
CDAI	Clinical disease activity index
CI	Confidence interval
CRP	C-reactive protein
csDMARDs	Conventional synthetic disease-modifying anti-rheumatic drugs
DIP	Distal interphalangeal joints
DMARDs	Disease-modifying anti-rheumatic drugs
ESR	Erythrocyte sedimentation rate
GCs	Glucocorticoids
HAQ-DI	Health assessment questionnaire disability index
HAQ	Health assessment questionnaire
IL-6i	Interleukin-6 inhibitors
JE	Joint erosion
JSN	Joint space narrowing
JAKi	Janus kinase inhibitors
mTSS	Modified total Sharp score
MCP	Metacarpophalangeal joints
OR	Odds ratio
PIP	Proximal interphalangeal joints
Pain VAS	Pain visual analogue scale
PrGA	Provider global assessment of disease activity
PtGA	Patient global assessment of disease activity
RA	Rheumatoid arthritis
RJD	Radiographic joint damage
RF	Rheumatoid factor
SD	Standard deviation
tsDMARDs	Targeted-synthetic disease-modifying anti-rheumatic drugs
TNFi	Tumor necrosis factor inhibitors
