# Supplementary material for: Association of Biologic/Targeted-Synthetic DMARDs with a Lower Prevalence of Hand Joint Deformity in Rheumatoid Arthritis: A Cross-Sectional Real-World Study
Source: Medicina (Kaunas). 2026 Jan 23;62(2):241. doi: 10.3390/medicina62020241 (PMC12942569; doi:10.3390/medicina62020241)
Supplement: Supplementary file 1 [file medicina-62-00241-s001.zip › medicina-4066842-supplementary.pdf]

Supplementary Materials

Appendix S1. Questions related to hand function on the Stanford health assessment questionnaire (HAQ).

Are you able to:
Component of dressing and grooming:
HAQ 1: Dress yourself, including managing fasteners?
HAQ 2: Comb your hair and do your own makeup?
Component of eating:
HAQ 3: Hold a chopstick?
HAQ 4: Lift a full cup or glass to your mouth?
HAQ 5: Open a new milk carton?
Component of grip:
HAQ 6: Turn locks on and off?
HAQ 7: Open jars which have been previously opened?
HAQ 8: Turn taps on and off?

Supplemental Table S1. Comparison of demographic, clinical, and hand joint deformity characteristics between RA patients in normal and elevated ESR/CRP groups

**Characteristics**	**In normal ESR and CRP (*n* = 370)**	**In elevated ESR/CRP (*n* = 713)**	***p***
Age, years	49.3±12.9	54.3±11.8	<0.001
Female, *n* (%)	321(86.8)	577(80.9)	0.016
Disease duration, years	5(2, 10)	6(2, 11)	0.285
Positive RF, *n* (%)	213(57.6)	515(72.2)	<0.001
Positive ACP A, *n* (%)	235(63.5)	482(67.6)	0.177
ESR (mm/h)	11(7, 16)	40(26, 63)	<0.001
CRP (mg/L)	3(3, 3)	7(3, 18)	<0.001
CDAI	5(0, 11)	12(5, 21)	<0.001
CDAI remission, *n* (%)	146(39.5)	121(17.0)	<0.001
Decreased grip strength, *n* (%)	216(58.4)	565(79.2)	<0.001
HAQ hand score	0.00(0.00, 0.13)	0.00(0.00, 0.38)	<0.001
HAQ hand disability, *n* (%)	93(25.1)	331(46.4)	<0.001
RJD, *n* (%)	163(44.1)	401(56.2)	<0.001
Hand joint deformity	78(21.1)	197(27.6)	0.019
Number of deformed joints	0(0, 0)	0(0, 1)	0.005
Number of deformed joints			
$1 \le n \le 3$	35 (9.5)	67 (9.4)	0.973
$4 \le n \le 5$	17 (4.6)	30 (4.2)	0.767
$6 \le n \le 10$	16 (4.3)	53 (7.4)	0.047
$11 \le n \le 15$	7 (1.9)	16 (2.2)	0.703
$16 \le n \le 20$	2 (0.5)	23 (3.2)	0.005
$21 \le n \le 28$	1 (0.3)	8 (1.1)	0.266
Location of hand joint deformity			

MCP	21 (5.7)	91 (12.8)	<0.001
MCP I	4 (1.1)	9 (1.3)	0.793
MCP II	9 (2.4)	66 (9.3)	<0.001
MCP III	11 (3.0)	64 (9.0)	<0.001
MCP IV	8 (2.2)	66 (9.3)	<0.001
MCP V	8 (2.2)	68 (9.5)	<0.001
PIP	67 (18.1)	156 (21.9)	0.145
PIP I	13 (3.5)	29 (4.1)	0.654
PIP II	18 (4.9)	65 (9.1)	0.013
PIP III	34 (9.2)	92 (12.9)	0.071
PIP IV	29 (7.8)	89 (12.5)	0.020
PIP V	41 (11.1)	99 (13.9)	0.192
DIP	42(11.4)	113 (15.8)	0.045
DIP II	13 (3.5)	56 (7.9)	0.006
DIP III	28 (7.6)	69 (9.7)	0.249
DIP IV	25 (6.8)	67 (9.4)	0.139
DIP V	23 (6.2)	72 (10.1)	0.032
Type of hand joint deformity			
Ulnar deviation of MCP II–V	12 (3.2)	75 (10.5)	<0.001
Boutonnniere fingers II–V	19 (5.1)	55 (7.7)	0.111
Swan neck fingers II–V	19 (5.1)	54 (7.6)	0.129
Hyperflexion of MCP I	12 (3.2)	30 (4.2)	0.436
Z deformity of thumb	9 (2.4)	21 (2.9)	0.626
Hypereflxsion of PIP I	13 (3.5)	29 (4.1)	0.654
Hypereflxsion of PIP II–V	25 (6.8)	39 (5.5)	0.394
Hyperextension of PIP II–V	3 (0.8)	3 (0.4)	0.424
Subluxation of PIP II–V	12 (3.2)	26(3.6)	0.732
Hyperflextion of DIP II–V	5 (1.4)	14 (2.0)	0.467
Hyperextension of DIP II–V	3 (0.8)	2 (0.3)	0.238
Subluxation of DIP II–V	2 (0.5)	9 (1.3)	0.421

RF, rheumatoid factor; ACPA, anti-cyclic citrullinated peptide antibody; ESR, erythrocyte sedimentation rate; CRP, C-reactive protein; CDAI, Clinical Disease Activity Index; HAQ, Health Assessment Questionnaire; RJD, radiographic joint damage; MCP, metacarpophalangeal joint; PIP, proximal interphalangeal joint; DIP, distal Interphalangeal joint.

Supplemental Table S2. Comparison of clinical characteristics between RA patients with and without hand joint deformity, stratified by ESR/CRP status

**Characteristics**	**Normal ESR and CRP***			**Elevated ESR or CRP***		
	**Without deformity (*n* = 292)**	**With deformity (*n* = 78)**	***p***	**Without deformity (*n* = 516)**	**With deformity (*n* = 197)**	***p***
Age, years	48.9 ± 13.1	50.9 ± 12.2	0.218	53.7 ± 12.0	55.8 ± 11.2	0.040
Female, *n* (%)	248(84.9)	73(93.6)	0.045	413(80.0)	164(83.2)	0.329
Active smoking, *n* (%)	32(11.0)	8(10.3)	0.859	74(14.3)	28(14.2)	0.965

Disease duration, years	4(2, 8)	10(5, 14)	<0.001	4(1, 9)	10(6, 17)	<0.001
Positive RF, *n* (%)	163(55.8)	50(64.1)	0.189	372(72.1)	143(72.6)	0.895
Positive ACPA, *n* (%)	184(63.0)	51(65.4)	0.699	342(66.3)	140(71.1)	0.222
Core disease activity indicators						
Morning stiffness time, min	0(0, 1)	0(0, 1)	0.681	0(0, 14)	0(0, 10)	0.071
28TJC	1(0, 3)	1(0, 3)	0.463	3(0, 8)	3(1, 9)	0.293
28SJC	0(0, 1)	0(0, 2)	0.109	1(0, 4)	2(0, 5)	0.543
PtGA	1(0, 3)	2(0, 4)	0.040	3(1, 5)	4(2, 6)	<0.001
PrGA	1(0, 3)	2(0, 3)	0.070	2(1, 5)	3(2, 5)	<0.001
Pain VAS	1(0, 2)	2(0, 3)	0.186	2(1, 4)	3(1, 5)	0.099.
ESR (mm/h)	11(7, 16)	12(7, 15)	0.893	40(26, 64)	40(26, 63)	0.760
CRP (mg/L)	3(3, 3)	3(3, 3)	0.257	7(3, 19)	7(3, 16)	0.280
CDAI	4(0, 11)	6(1, 13)	0.071	11(4, 21)	14(6, 24)	0.007
CDAI remission, *n* (%)	125(42.8)	21(26.9)	0.011	98(19.0)	23(11.7)	0.020
Low disease activity, *n* (%)	82(28.1)	29(37.2)		133(25.8)	48(24.4)	
Middle disease activity, *n* (%)	59(20.2)	22(28.2)		166(32.2)	68(34.5)	
High disease activity, *n* (%)	26(8.9)	6(7.7)		119(23.1)	58(29.4)	
Functional indicators						
Decreased grip strength, *n* (%)	156(53.4)	60(76.9)	<0.001	394(76.4)	171(86.8)	0.002
HAQ hand score	0.00(0.00, 0.00)	0.00(0.00, 0.13)	0.476	0.00(0.00, 0.25)	0.13(0.00, 0.50)	0.033
HAQ hand disability, *n* (%)	71(24.3)	22(28.2)	0.482	229(44.4)	102(51.8)	0.077
Radiographic assessment						
mTSS	8(2, 23)	25(2, 48)	<0.001	13(4, 33)	32(12, 73)	<0.001
JSN	1(0, 7)	10(2, 21)	<0.001	4(0, 13)	14(4, 35)	<0.001
JE	5(1, 14)	11(5, 28)	<0.001	9(3, 20)	17(6, 39)	<0.001
RID, *n* (%)	111(38.0)	52(66.7)	<0.001	256(49.6)	145(73.6)	<0.001

RF, rheumatoid factor; ACPA, anti-cyclic citrullinated peptide antibody; 28TJC, 28-joint tender joint counts; 28SJC, 28-joint swollen joint counts; PtGA, patient global assessment of disease activity; PrGA, provider global assessment of disease activity; Pain VAS, pain visual analog scale; ESR, erythrocyte sedimentation rate; CRP, C-reactive protein; CDAI, Clinical Disease Activity Index; HAQ, Health Assessment Questionnaire; mTSS, modified total Sharp score; JSN, joint space narrowing; JE, joint erosion; RJD, radiographic joint damage.

Supplemental Table S3. Comparison of clinical characteristics between RA patients with and without hand joint deformity, stratified by ESR/CRP status, after propensity score matching disease duration

**Characteristics**	**After matching in normal ESR and CRP***			**After matching in elevated ESR or CRP***		
	**Without deformity (*n* = 94)**	**With deformity (*n* = 78)**	***p***	**Without deformity (*n* = 181)**	**With deformity (*n* = 197)**	***p***
Age, years	48.9 ± 14.6	50.9 ± 12.2	0.258	54.2 ± 12.0	55.8 ± 11.2	0.186
Female, *n* (%)	86(91.5)	73(93.6)	0.604	162(89.5)	164(83.2)	0.078
Active smoking, *n* (%)	8(8.5)	8(10.3)	0.695	19(10.5)	28(14.2)	0.274
Disease duration, years	9(4, 12)	10(5, 14)	0.570	10(7, 16)	10(6, 17)	0.963
Positive RF, *n* (%)	54(57.4)	50(64.1)	0.374	133(73.5)	143(72.6)	0.845

Positive ACPA, *n* (%)	53(56.4)	51(65.4)	0.229	121(66.9)	140(71.1)	0.376
Core disease activity indicators						
Morning stiffness time, min	0(0, 2)	0(0, 1)	0.705	0(0, 5)	0(0, 10)	0.722
28TJC	1(0, 2)	1(0, 3)	0.301	2(0, 6)	3(1, 9)	0.015
28SJC	0(0, 1)	0(0, 2)	0.246	1(0, 3)	2(0, 5)	0.042
PtGA	1(0, 3)	2(0, 4)	0.035	3(1, 5)	4(2, 6)	0.007
PrGA	1(0, 2)	2(0, 3)	0.096	2(1, 5)	3(2, 5)	0.002
Pain VAS	1(0, 2)	2(0, 3)	0.233	2(1, 4)	3(1, 5)	0.111
ESR (mm/h)	11(6, 17)	12(7, 15)	0.890	37(27, 59)	40(26, 63)	0.442
CRP (mg/L)	3(3, 3)	3(3, 3)	0.104	6(3, 15)	7(3, 16)	0.603
CDAI	4(0, 9)	6(1, 13)	0.070	10(4, 17)	14(6, 24)	0.001
CDAI remission, *n* (%)	38(40.4)	21(26.9)	0.063	35(19.3)	23(11.7)	0.039
Low disease activity, *n* (%)	33(35.1)	29(37.2)		54(29.8)	48(24.4)	
Middle disease activity, *n* (%)	17(18.1)	22(28.2)		63(34.8)	68(34.5)	
High disease activity, *n* (%)	6(6.4)	6(7.7)		29(16.0)	58(29.4)	
Functional indicators						
Decreased grip strength, *n* (%)	58(61.7)	60(76.9)	0.032	136(75.1)	171(86.8)	0.004
HAQ hand score	0.00(0.00, 0.13)	0.00(0.00, 0.13)	0.611	0.00(0.00, 0.25)	0.13(0.00, 0.50)	0.003
HAQ hand disability, *n* (%)	24(25.5)	22(28.2)	0.693	70(38.7)	102(51.8)	0.011
Radiographic assessment						
mTSS	10(4, 31)	25(8, 48)	0.006	23(9, 43)	32(12, 73)	0.003
JSN	3(0, 11)	10(2, 21)	<0.001	9(1, 20)	14(4, 35)	<0.001
JE	8(2, 21)	11(5, 28)	0.071	13(6, 26)	17(6, 39)	0.028
RID, *n* (%)	42(44.7)	52(66.7)	0.007	114(63.0)	145(73.6)	0.071

Values of continuous variables were presented as mean and standard deviation or median with interquartile range according to distributions, and categorical variables were presented as numbers and percentages. Comparisons among three groups by the one-way ANOVA test or chi-squared test. A *P*-value < 0.05 was defined as a significant difference, which was shown in bold.

*Propensity score matching between RA patients without hand joint deformity and with hand joint deformity, and controls with 1:1 matching by disease duration.

RF, rheumatoid factor; ACPA, anti-cyclic citrullinated peptide antibody; 28TJC, 28-joint tender joint counts; 28SJC, 28-joint swollen joint counts; PtGA, patient global assessment of disease activity; PrGA, provider global assessment of disease activity; Pain VAS, pain visual analog scale; ESR, erythrocyte sedimentation rate; CRP, C-reactive protein; CDAI, Clinical Disease Activity Index; HAQ, Health Assessment Questionnaire; mTSS, modified total Sharp score; JSN, joint space narrowing; JE, joint erosion; RJD, radiographic joint damage.

Supplemental Table S4. Comparison of hand joint deformity characteristics by use of b/tsDMARDs in matched RA patients

**Characteristics**	**Conventional medicine (*n* = 188)**	**TNFi (*n* = 52)**	**IL-6i (*n* = 42)**	**JAKi (*n* = 91)**	***p*[a]**
Hand joint deformity, *n* (%)	116(61.7)	15(28.8)[b]	16(38.1)[b]	20(22.0)[b]	<0.001

Type of deformity					
Boutonniere fingers II–V, *n* (%)	27(14.4)	6(11.5)	2(4.8)	5(5.5)	0.087
Swan neck fingers II–V, *n* (%)	30(16.0)	5(9.6)	4(9.5)	4(4.4)[b]	0.038
Z deformity of thumb, *n* (%)	9(4.8)	0(0)	2(4.8)	4(4.4)	0.301
Ulnar deviation of MCP II–V, *n* (%)	36(19.1)	6(11.5)	5(11.9)	4(4.4)[b]	0.007
Hyperflexion of MCP I, *n* (%)	5(2.7)	0(0)	2(4.8)	0(0)	0.115
Hyperflexion of PIP I, *n* (%)	3(1.6)	1(1.9)	0(0)	2(2.2)	0.796
Hyperflexion of PIP II–V, *n* (%)	33(17.6)	3(5.8)[b]	4(9.5)	0(0)[b, c, d]	<0.001
Hyperextension of PIP II–V, *n* (%)	3(1.6)	0(0)	0(0)	1(1.1)	0.642
Subluxation of PIP II–V, *n* (%)	18(9.6)	2(3.8)	3(7.1)	4(4.4)	0.383
Hyperflexion of DIP II–V, *n* (%)	7(3.7)	0(0)	1(2.4)	2(2.2)	0.435
Hyperextension of DIP II–V, *n* (%)	4(2.1)	0(0)	0(0)	0(0)	0.232
Subluxation of DIP II–V, *n* (%)	5(2.7)	1(1.9)	1(2.4)	0(0)	0.383

DMARDs, disease-modifying anti-rheumatic drugs; Conventional medicine, conventional DMARDs and/or GCs; b/ts, biological or targeted-synthetic disease-modifying antirheumatic drugs; RF, rheumatoid factor; ACPA, anti-cyclic citrullinated peptide antibody; ESR, erythrocyte sedimentation rate; CRP, C-reactive protein; CDAI, Clinical Disease Activity Index; HAQ, Health Assessment Questionnaire; RJD, radiographic joint damage; MCP, metacarpophalangeal joint; PIP, proximal interphalangeal joint; DIP, distal interphalangeal joint.

[a] Comparison in four groups by Chi-square test.

[b] Compared with patients treatment with Conventional medicine in Bonferroni correction, $p < 0.0083$.

[c] Compared with patients treatment with Conventional medicine+TNFi in Bonferroni correction, p < 0.0083.

[d] Compared with patients treatment with Conventional medicine+IL-6i in Bonferroni correction, $p < 0.0083$.

Supplemental Table S5. Comparison of patient characteristics and hand joint deformity by use of MTX and b/tsDMARDs in matched RA patients

**Characteristics**	**Conventional medicine (n = 188)**		**Conventional medicine+b/ts (n = 188)**		p^a
	**Non-MTX (*n* = 42)**	**MTX (*n* = 146)**	**Non-MTX + b/ts (*n* = 35)**	**MTX + b/ts (*n* = 153)**	
Hand joint deformity, *n* (%)	28(66.7)	88(60.3)	10(28.6)[b, c]	41(26.8)[b, c]	<0.001
Type of deformity					
Boutonniere fingers II–V, *n* (%)	8(19.0)	19(13.0)	5(14.3)	8(5.2)[b, c]	0.023
Swan neck fingers II-V, *n* (%)	3(7.1)	27(18.5)	3(8.6)	10(6.5)[c]	0.009
Z deformity of thumb, *n* (%)	1(2.4)	8(5.5)	1(2.9)	5(3.3)	0.696
Ulnar deviation of MCP II–V, *n* (%)	10(23.8)	26(17.8)	1(2.9)[b, c]	14(9.2)[b, c]	0.005
Hyperflexion of MCP I, *n* (%)	0(0)	5(3.4)	0(0)	2(1.3)	0.191
Hyperflexion of PIP I, *n* (%)	0(0)	3(2.1)	1(2.9)	2(1.3)	0.592
Hyperflexion of PIP II–V, *n* (%)	7(16.7)	26(17.8)	0(0)[b, c]	7(4.6)[b, c]	<0.001
Hyperextension of PIP II–V, *n* (%)	0(0)	3(2.1)	0(0)	1(0.7)	0.391
Subluxation of PIP II–V, *n* (%)	4(9.5)	14(9.6)	3(8.6)	6(3.9)	0.216
Hyperflexion of DIP II–V, *n* (%)	2(4.8)	5(3.4)	0(0)	3(2.0)	0.378

Hyperextension of DIP II–V, *n* (%)	1(2.4)	3(2.1)	0(0)	0(0)	0.133
Subluxation of DIP II–V, *n* (%)	3(7.1)	2(1.4)	1(2.9)	1(0.7)	0.124

MTX, methotrexate; DMARDs, disease-modifying anti-rheumatic drugs; Conventional medicine, conventional DMARDs and/or GCs; b/ts, biological or targeted-synthetic disease-modifying antirheumatic drugs; RF, rheumatoid factor; ACPA, anti-cyclic citrullinated peptide antibody; ESR, erythrocyte sedimentation rate; CRP, C-reactive protein; CDAI, Clinical Disease Activity Index; HAQ, Health Assessment Questionnaire; RJD, radiographic joint damage; MCP, metacarpophalangeal joint; PIP, proximal interphalangeal joint; DIP, distal interphalangeal joint.

[a] Comparison in four groups by Chi-square test.

[b] Compared with patients treatment without MTX in Bonferroni correction, $p < 0.0083$.

[c] Compared with patients treatment with MTX in Bonferroni correction, $p < 0.0083$.

[d] Compared with patients treatment without MTX+b/tsDMARDs in Bonferroni correction, $p < 0.0083$.

Supplemental Table **S**6. Association between subgroups of MTX and b/tsDMARDs with hand joint deformity in patients with RA

**Subgroups of MTX and b/ts**	**Univariate logistic regression**		**Multivariate logistic regression**	
	**OR (95% CI)**	***p***	**AOR (95% CI)***	***p***
Non-MTX + non-b/ts	Ref	NA	Ref	NA
MTX + non-b/ts	0.759 (0.368–1.562)	0.453	0.958 (0.418–2.199)	0.920
Non-MTX + b/ts	0.200 (0.075–0.530)	0.001	0.340 (0.112–1.029)	0.056
MTX + b/ts	0.183 (0.088–0.382)	<0.001	0.180 (0.079–0.412)	<0.001

MTX, methotrexate; b/ts, biological or targeted-synthetic DMARDs; Ref, reference; NA, not applicable.

* Ajusted for age, sex, disease duration, active smoking, positive RF and ACPA, morning stiffness, Pain VAS, ESR, CRP, CDAI, mTSS.
